# A benchmark dataset for Hydrogen Combustion

**DOI:** 10.1038/s41597-022-01330-5

**Published:** 2022-05-17

**Authors:** Xingyi Guan, Akshaya Das, Christopher J. Stein, Farnaz Heidar-Zadeh, Luke Bertels, Meili Liu, Mojtaba Haghighatlari, Jie Li, Oufan Zhang, Hongxia Hao, Itai Leven, Martin Head-Gordon, Teresa Head-Gordon

**Affiliations:** 1 Kenneth S. Pitzer Theory Center and Department of Chemistry, University of California, Berkeley, CA USA; 2 Chemical Sciences Division, Lawrence Berkeley National Laboratory, Berkeley, CA USA; 3 Theoretical Physics and Center for Nanointegration Duisburg-Essen (CENIDE), University of Duisburg-Essen, 47048 Duisburg, Germany; 4 Department of Chemistry, Queen’s University, Kingston, Ontario K7L 3N6 Canada; 5 Department of Chemistry, Beijing Normal University, Beijing, 100875 China; 6 Departments of Bioengineering and Chemical and Biomolecular Engineering, University of California, Berkeley, CA USA

**Keywords:** Method development, Databases

## Abstract

The generation of reference data for deep learning models is challenging for reactive systems, and more so for combustion reactions due to the extreme conditions that create radical species and alternative spin states during the combustion process. Here, we extend intrinsic reaction coordinate (IRC) calculations with *ab initio* MD simulations and normal mode displacement calculations to more extensively cover the potential energy surface for 19 reaction channels for hydrogen combustion. A total of ∼290,000 potential energies and ∼1,270,000 nuclear force vectors are evaluated with a high quality range-separated hybrid density functional, *ω*B97X-V, to construct the reference data set, including transition state ensembles, for the deep learning models to study hydrogen combustion reaction.

## Abstract (summary)

**Table:** 

Measurement(s)	ab initio energies and forces of hydrogen combustion
Technology Type(s)	density functional theory • ab initio molecular dynamics • normal modes
Factor Type(s)	cartesian coordinates

## Background & Summary

The expectation behind training deep learning models to predict molecular energies and atomic forces of molecules is the requirement of large data sets. However, very recently it has become recognized that deep learning methods that are designed with rotationally equivariant operators offer a significant reduction in data needed for training relative to invariant ML models^1–4^, and often outcompete even kernal methods that have traditionally been considered advantageous due to their low data requirements^5^. However, the promise in regards equivariant deep learning models must be further validated by construction of more challenging data sets than encountered up until now. For example, the recent SN2 data set provides reference energy and forces for more than 450,000 structures calculated using Density Functional Theory (DFT), but ultimately is data on highly similar individual reactions of methyl halides with one of four substituted halogens, F, Cl, Br, and I^6^.

Capturing the energy release in hydrogen combustion is a proposed energy solution for zero CO_2_ emissions, and many of the elementary reactions of H_2_ combustion are also present in other types of fuel generation^7^. Under realistic reaction conditions of very high temperature and high pressure make it extremely difficult to study H_2_ combustion reactions experimentally. Because hydrogen combustion is difficult to study experimentally under these extremes^8^, theoretical models must play an active role in filling the breach, but fundamentally relies on an accurate potential energy model of not only the elementary reactions^9^ but the excursions away from the reaction coordinate.

Hydrogen combustion, despite being the simplest combustion system, is nonetheless still quite chemically complicated because it can encounter one or more 19 reaction channels during the combustion event depending on the physical conditions of high temperatures and pressures^8^. This compounds the need for high quality data that is expensive to generate given the need for extensive sampling and the presence of metastable points such as transition states. For non-reacting chemical systems, conventional MD simulations are well-suited for generating a large number of configurations, which are then used as input into single point quantum-chemical energy and force calculations^10–12^. However, for reactive systems, conventional force-field based MD simulations are not useful as they don’t allow breaking and forming of chemical bonds. Recent work has attempted to address this deficiency through graph-based methods that generate reference data for reactive systems^13,14^, but they are also prone to produce large numbers of specious chemical states and unrealistic intermediates such as highly unstable radicals. Therefore fully *ab initio* sampling methods are a necessity for creation of the many molecular fragments involved in combustion chemistry, including the presence of stable and unstable intermediates, high energy transition states, and a variety of product molecules that can be formed during the reaction that is dependent on the reactive channel^8,9,15–18^.

Our goal here is to characterize the potential energy surface (PES) of hydrogen combustion through the reaction channels proposed by Li *et al*.^19^ using a systematic approach in *ab initio* data generation that samples off the intrinsic reaction coordinate (IRC). This study provides a data set of ∼290,000 potential energies and ∼1,270,000 nuclear force vectors for structures that are sampled near and far from the IRC for 19 hydrogen combustion sub-reactions, some of which are barrierless transitions, others are dominated by large activation barriers, and even reactions involving changes in spin state^19^. This data set offers a new ML benchmark set that allows systematic investigation of data reduction when using emerging equivariant deep learning model, as well as being of interest in its own right as a source of data for machine learning of energy and forces that drive an MD engine for combustion under extreme thermodynamic conditions.

## Methods

We have used fully *ab initio* methods for sampling 19 reactive channels for hydrogen combustion as summarized in Table 1. For each reaction we used the *ω*B97X-V DFT functional^20^ with the cc-pVTZ basis set. All calculations were performed as unrestricted open shell, using an ultrafine integration grid of 99 radial points and 590 angular points, with an SCF convergence of $1{0}^{-8}$ using the GDM method^21^. All potential energies for each configuration of the 19 reactions are reported as ΔE

1 $$\Delta E={E}_{total}-\sum _{i}{E}_{atom},$$

using the atomic energies E_H_ = −0.5004966690 a.u. and E_o_ = −75.0637742413 a.u., and with ΔE converted to units of kcal/mole. All calculations were performed using the Q-Chem program^22,23^.

**Table 1:** Data Summary for the Potential Energy Surface of Hydrogen Combustion.

No. Reaction	Atoms	IRC	MD simulations	Normal mode	Total energies	Total forces
**Association/Dissociation**						
5. ${{\rm{H}}}_{2}\to 2{\rm{H}}$	2	53			53	318
6. ${{\rm{O}}}_{2}\to 2{\rm{O}}$	2	71			71	426
7. ${\rm{OH}}\to {\rm{O}}+{\rm{H}}$	2	71			71	426
8. ${\rm{H}}+{\rm{OH}}\to {{\rm{H}}}_{{\rm{2}}}{\rm{O}}$	3	137	10000	5754	15891	143019
9. ${\rm{H}}+{{\rm{O}}}_{2}\to {{\rm{HO}}}_{2}$	3	60	10000	2520	12580	113220
15. ${{\rm{H}}}_{2}{{\rm{O}}}_{2}\to 2{\rm{OH}}$	4	105	10000	8820	18925	227100
**Substitution**						
16. ${{\rm{H}}}_{2}{{\rm{O}}}_{2}+{\rm{H}}\to {{\rm{H}}}_{2}{\rm{O}}+{\rm{OH}}$	5	81	10000	10206	20287	304305
**O-transfer**						
1. ${\rm{H}}+{{\rm{O}}}_{2}\to {\rm{OH}}+{\rm{O}}$	3	58	10000	3248	13306	119754
11. ${{\rm{HO}}}_{2}+{\rm{H}}\to 2{\rm{OH}}$	4	94	10000	7896	17990	215880
12. ${{\rm{HO}}}_{2}+{\rm{O}}\to {\rm{OH}}+{{\rm{O}}}_{2}$	4	49	10000	4116	14165	169980
**H-transfer**						
2. ${\rm{O}}+{{\rm{H}}}_{2}\to {\rm{OH}}+{\rm{H}}$	3	29	10000	1624	11653	104877
3. ${{\rm{H}}}_{2}+{\rm{OH}}\to {{\rm{H}}}_{2}{\rm{O}}+{\rm{H}}$	4	336	10000	30492	40828	489936
4. ${{\rm{H}}}_{2}{\rm{O}}+{\rm{O}}\to 2{\rm{OH}}$	4	51	10000	4284	14335	172020
10. ${{\rm{HO}}}_{2}+{\rm{H}}\to {{\rm{H}}}_{2}+{{\rm{O}}}_{2}$	4	58	10000	4872	14930	179160
13. ${{\rm{HO}}}_{2}+{\rm{OH}}\to {{\rm{H}}}_{2}{\rm{O}}+{{\rm{O}}}_{2}$	5	51	10000	6426	16477	247155
14. $2{{\rm{HO}}}_{2}\to {{\rm{H}}}_{2}{{\rm{O}}}_{2}+{{\rm{O}}}_{2}$	6	71	10000	11928	21999	395982
17. ${{\rm{H}}}_{2}{{\rm{O}}}_{2}+{\rm{H}}\to {{\rm{HO}}}_{2}+{{\rm{H}}}_{2}$	5	58	10000	7308	17366	260490
18. ${{\rm{H}}}_{2}{{\rm{O}}}_{2}+{\rm{O}}\to {{\rm{HO}}}_{2}+{\rm{OH}}$	5	55	10000	6930	16985	254775
19. ${{\rm{H}}}_{2}{{\rm{O}}}_{2}+{\rm{OH}}\to {{\rm{H}}}_{2}{\rm{O}}+{{\rm{HO}}}_{2}$	6	74	10000	12432	22506	405108
**Total**					290418	1267977

Tabulated are the number of structures generated for each hydrogen combustion reaction channel using different methods: IRC, normal mode displacements, and MD simulations at various temperatures. All 19 reaction channels are classified into four mechanistic groups: association/dissociation, substitution, O-transfer and H-transfer. For each configuration, energies and nuclear force vectors were computed and their numbers are tabulated.

We have organized the PES data into four categories that classify the reaction mechanism involved in the elementary steps for each reactive channel: association/dissociation reactions (channels 5-9 and 15), substitution reactions (channel 16), oxygen transfer (channels 1, 11, and 12), and hydrogen transfer (channels 2-4, 10, 13, 14, 17–19). We have kept the same numbering scheme as Li and co-workers^19^ in these categories so that readers can refer back to any particular IRC of that work if desired.

The PES for each reaction channel are visualized by means of two collective variables of coordination numbers (CN) represented by

2 $$CN=\sum _{i}\frac{2.0}{1+{\rm{\exp }}\left(\sigma \ast \left({r}_{i}-{r}_{0,i}\right)\right)},$$

where ${r}_{0}$ is the equilibrium distance and $\sigma =3.0$ controls the sharpness of the function. Reaction channels 5–7 involve only two atoms, and thus only a 1-D distance scan is performed.

Finally, we developed a strategy for extensive sampling of the PES for the 19 reaction channels for hydrogen combustion as follows:

1. *Transition States and IRCs*. Approximate TS structures were found using the freezing string method^24,25^, and refined by the partitioned-rational function optimization eigenvector following method (P-RFO)^26^. An IRC scan is then generated, and vibrational frequency analysis was performed to confirm that reactants and products have no imaginary frequencies and the TS has only one imaginary frequency. As the IRC configurations connect the minimum energy pathway, and therefore span a meaningful fraction of the configurational space of a given reaction, they serve as useful starting geometries for systematic normal mode displacements and stochastic generation of structures using AIMD at finite temperatures to explore the PES for each reaction channel in more detail.
2. *AIMD Simulations*. We employed AIMD simulations to sample configurations around the IRC structures using the TS as the initial configuration for each of the reaction channels. The AIMD simulations were performed at four different high temperatures by initializing the Maxwell-Boltzmann distribution of velocities at temperatures of 500 K, 1000 K, 2000 K and 3000 K. Furthermore at each temperature three different simulation timescales are performed using a 1.21 fs (1.au.) time step: 10 independent (i.e. reinitialized velocities) long simulations of 121 fs, 20 independent short trajectories of 60.5 fs, and finally 25 very short simulations of 24.2 fs. In summary, the AIMD calculations yielded a total of 10000 configurations along with their potential energies and nuclear forces for each reaction channel (see Table 1).
3. *Normal Mode Displacements*. Systematic normal mode displacements along the IRC is performed. Starting from each IRC structure, the frequencies were calculated and all atoms were displaced along each normal mode (NM) with a $\pm 0.01$, $\pm 0.025$, $\pm 0.05$, $\pm 0.075$, $\pm 0.1$, $\pm 0.125$, and ±0.15. increment. These sampled structures that compress or expand the IRC structures help to diversify the AIMD geometries for each reaction, yielding ∼ 127,000 configurations as summarized in Table 1. The IOData Python library was used for parsing the Q-Chem output files in generating these geometries^27^.

## Technical Validation

**Fig. 1:**
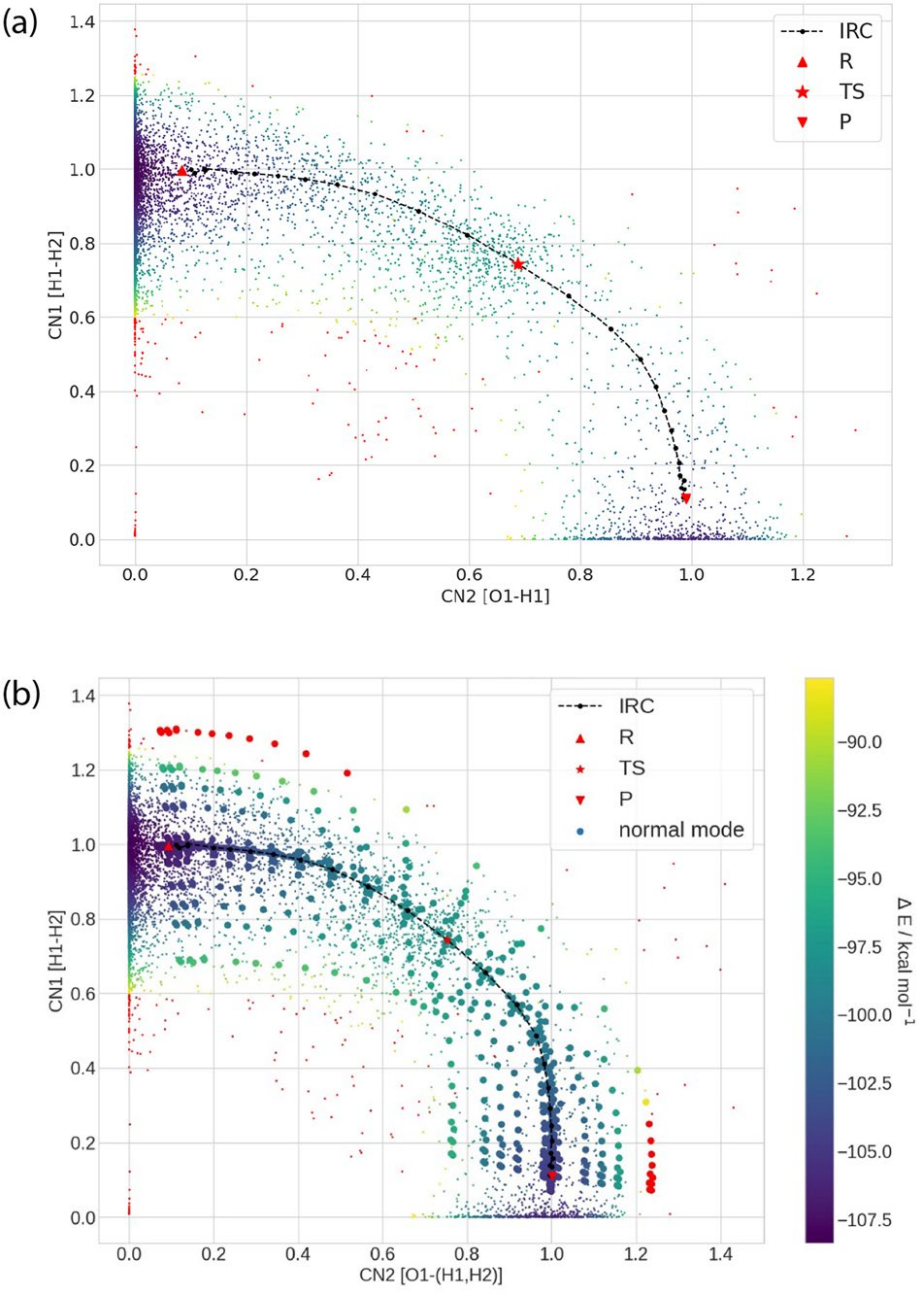
Potential energy surface for the hydrogen transfer reaction 2 (${\rm{O}}+{{\rm{H}}}_{2}\to {\rm{OH}}+{\rm{H}}$). (**a**) showing IRC and AIMD sample data only and (**b**) including normal mode data. CN1 represents the breaking of the H-H bond and CN2 represents the formation of the O-H bond. All energies are reported with respect to the atomization energies as given in Eq. (1) in units of kcal/mol. The red dots on the energy surface are configurations with energies larger than 10 kcal/mol of the energy of the TS structure. The points denoted with R, TS and P are corresponds to the reactant, transition state and product, respectively.

Figure 1 provides a representative *ab initio* sampling of one of the hydrogen transfer reactions, ${\rm{O}}+{H}_{2}\to OH+H$, in which two collective coordinates reasonably capture the potential energy surface of this reaction channel. Upon analyzing the AIMD generated geometries and their energies, it is noticed that both the reactant and product endpoint regions are well sampled (Fig. 1(a)). However, near the transition state or in regions of high slope on the potential energy surface, data points from the AIMD simulations are more sparse. The addition of normal mode displacement points greatly improves sampling the configuration space of the PES along the IRC path (Fig. 1(b)).

**Fig. 2:**
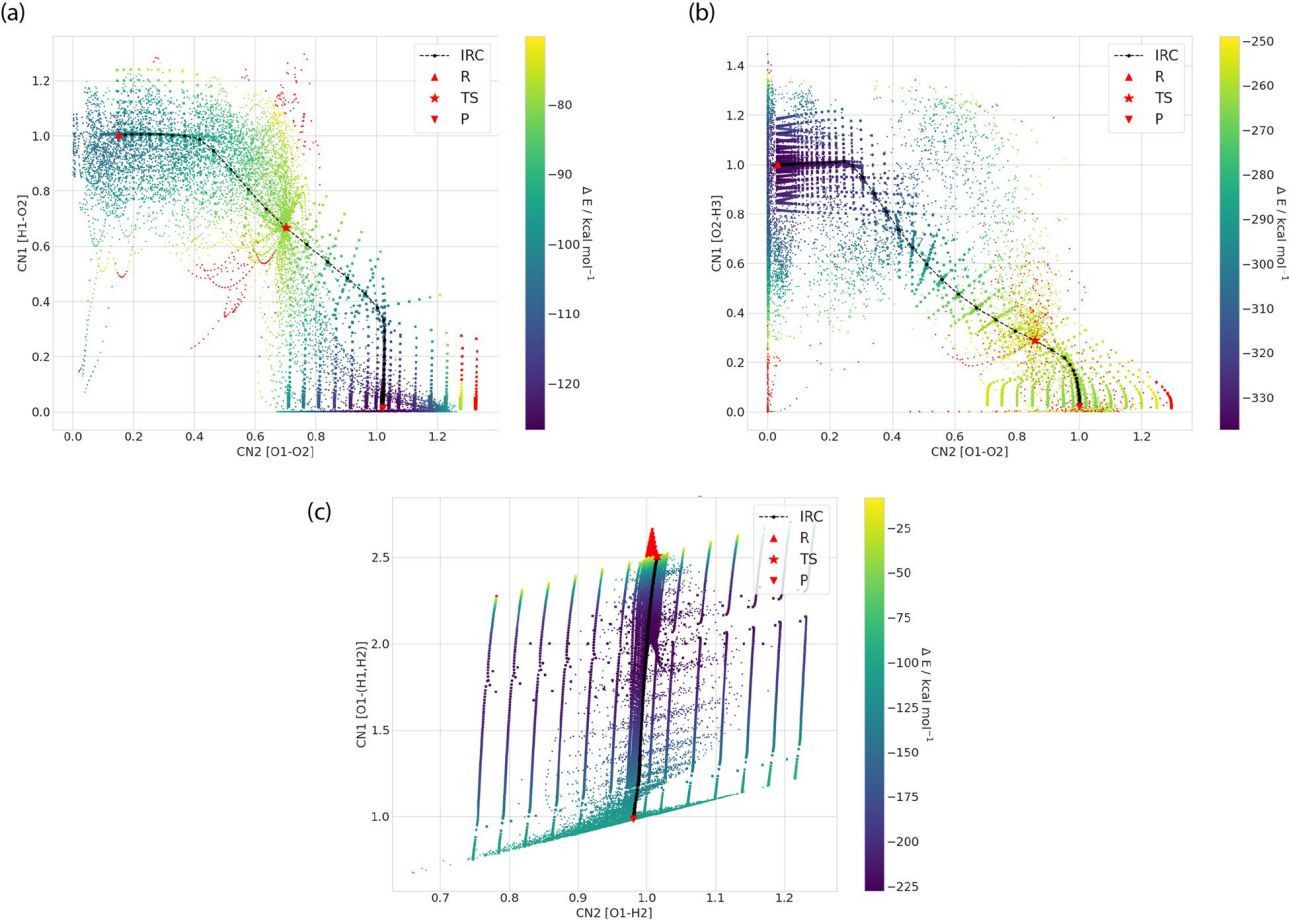
Representative potential energy surfaces for oxygen transfer, association, and substitution reactions along two reaction coordinates CN1 and CN2. (**a**) oxygen transfer reaction 1 (${\rm{H}}+{{\rm{O}}}_{2}\to {\rm{O}}{\rm{H}}+{\rm{O}}$), (**b**) association reaction 8 (${\rm{H}}+{\rm{O}}{\rm{H}}\to {{\rm{H}}}_{2}{\rm{O}}$), and (**c**) substitution reaction 16 (${{\rm{H}}}_{2}{{\rm{O}}}_{2}+{\rm{H}}\to {{\rm{H}}}_{2}{\rm{O}}+{\rm{OH}}$). Each CN represents the formation or breaking of respective bond involved in the reaction process mentioned in the axes.

Figure 2 shows that the AIMD and NM calculations are complementary for sampling different areas away from the IRC, particularly evident for reaction channel 1 involving oxygen transfer (Fig. 2(a)), reaction 8 that probes the association reaction mechanism (Fig. 2(b)), and for reaction channel 16 pertaining to a substitution mechanism (Fig. 2(c)). In all cases the use of two collective coordinates is sufficient to capture the IRC and its AIMD and NM extensions, borne out in the supplementary information Figures S1–S4 that provides the potential energy surfaces generated for the remaining reaction channels for these classes of hydrogen combustion reactions.

**Fig. 3:**
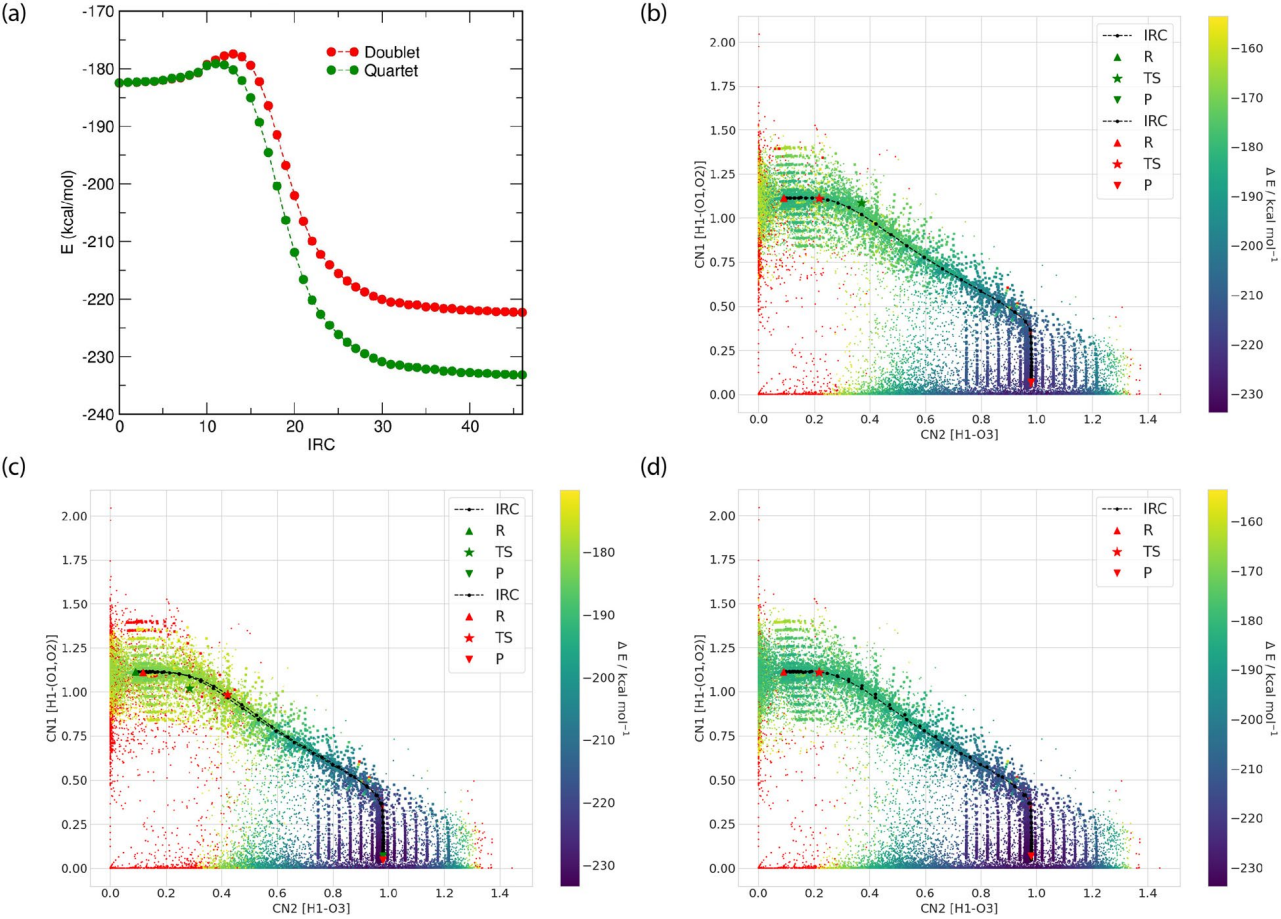
The changes in the PES for reaction channel 12 involving changes in spin state. (**a**) the spin cross over between the two closely spaced doublet and quartet spin state energy levels around the reactant region with widening differences progressing to product. (**b**) the IRC path defined by the doublet energy but geometries from the quartet (green), and from the doublet energy and geometries (red). (**c**) the IRC path defined by the quartet energy but geometries from the doublet (green) and from the quartet energies and geometries (red). (**d**) Resultant PES obtained reaction channel 12 (${{\rm{HO}}}_{2}+{\rm{O}}\to {\rm{OH}}+{{\rm{O}}}_{2}$) by choosing the minimum energy between the two spin states. Each CN represents the formation or breaking of respective bond involved in the reaction process mentioned in the axes.

Figure 3 shows the nature of the alternative potential energy surfaces that are represented by the changes in spin state from doublet to quartet for the oxygen transfer reaction channel 12. Figure 3(a) shows that the energy difference between the two spin states is very small near the reactant, less than 0.2 kcal/mol, but favors the quartet state substantially around the product. Figure 3(b) plots the IRC using either the doublet or quartet spin state energies using the quartet spin state static structures, and similarly Fig. 3(c) represents the two spin state energies using the doublet energy configurations. Figure 3(d) shows the minimum energy of the two spin states along a single generated IRC. These differences indicate that while the geometric effects may be small, the electronic energy differences between spin states are significant. In the supplementary information we also provides the potential energy surfaces generated for reaction channel 6 which also undergoes a spin state change.

In summary, we generated high quality DFT data for hydrogen combustion reaction channels using range separated hybrid meta-GGA functional *ω*B97X-V with the cc-pVTZ basis set. This level of theory is considered highly accurate for thermochemistry and reactive barriers^28,29^, and the IRC profiles compared against the gold standard CCSD(T)/cc-pVTZ methods determined very small errors with the DFT level of theory^7^. This work moves beyond benchmarks such as the IRC for H_2_ combustion by extensive sampling off the reaction coordinate using *ab initio* MD simulation and normal mode analysis for each of the 19 reaction channels. Furthermore, we also consider multiple spin states of the species formed in the hydrogen combustion process. This high quality data is now available to benchmark deep learning models for chemical reactivity, and as a model of the PES for generating kinetic models of H_2_ combustion, especially at high pressure.

## Data Records

All data can be found in the figshare repository. For each reaction channel the IRC, AIMD and NM generated configurations and corresponding energies and atomic forces are provided in.npz file format; for reaction channel 5, 6 and 7 only IRC generated data are provided as discussed above. Each .npz file contains six keys including, “R” (atomic Cartesian coordinates), “Z” (atomic numbers), “N” (number of atoms), “ΔE” (reference potential energy), “F” (atomic force vectors), and “RXN” (reaction number). All the atomic position are in Å and energy and force vectors are provided in kcal/mol and kcal/mol/Å, respectively. Reaction channels such as 6 and 12 involve nuclear spin changes during the reaction, and therefore IRC calculations are performed for both spin states, with the data sorted to either (1) retain energies and forces consistent with one spin state, or (2) retaining the lowest energy spin state along the IRC for each channel. Furthermore, for reactions 6 and 12 two sets of data are provided namely 06a/06b and 12a/12b corresponding to two different spin states involved in the reaction process.

## Usage Notes

The data set contains 19 folders corresponding to each of the reaction channels. Each reaction channel has three.npz files storing the geometries and corresponding potential energies energies and atomic force vectors obtained from IRC, AIMD and NM simulations separately. Each.npz file contains the “R” (atomic Cartesian coordinates), “Z” (atomic numbers), “N” (number of atoms), “ΔE” (reference potential energy), “F” (atomic forces), and “RXN” (reaction number) keys and the corresponding values for each configuration.

## Supplementary information

Fig S1

Fig S2

Fig S3

Fig S4

## Data Availability

All the data and python scripts used to generate coordination number based PES surface to analyze the data for each reaction channel is provided at 10.6084/m9.figshare.19601689^30^.
