# Supplementary figures and images for: A benchmark dataset for Hydrogen Combustion
Source: Sci Data. 2022 May 17;9:215. doi: 10.1038/s41597-022-01330-5 (PMC9114378; doi:10.1038/s41597-022-01330-5)

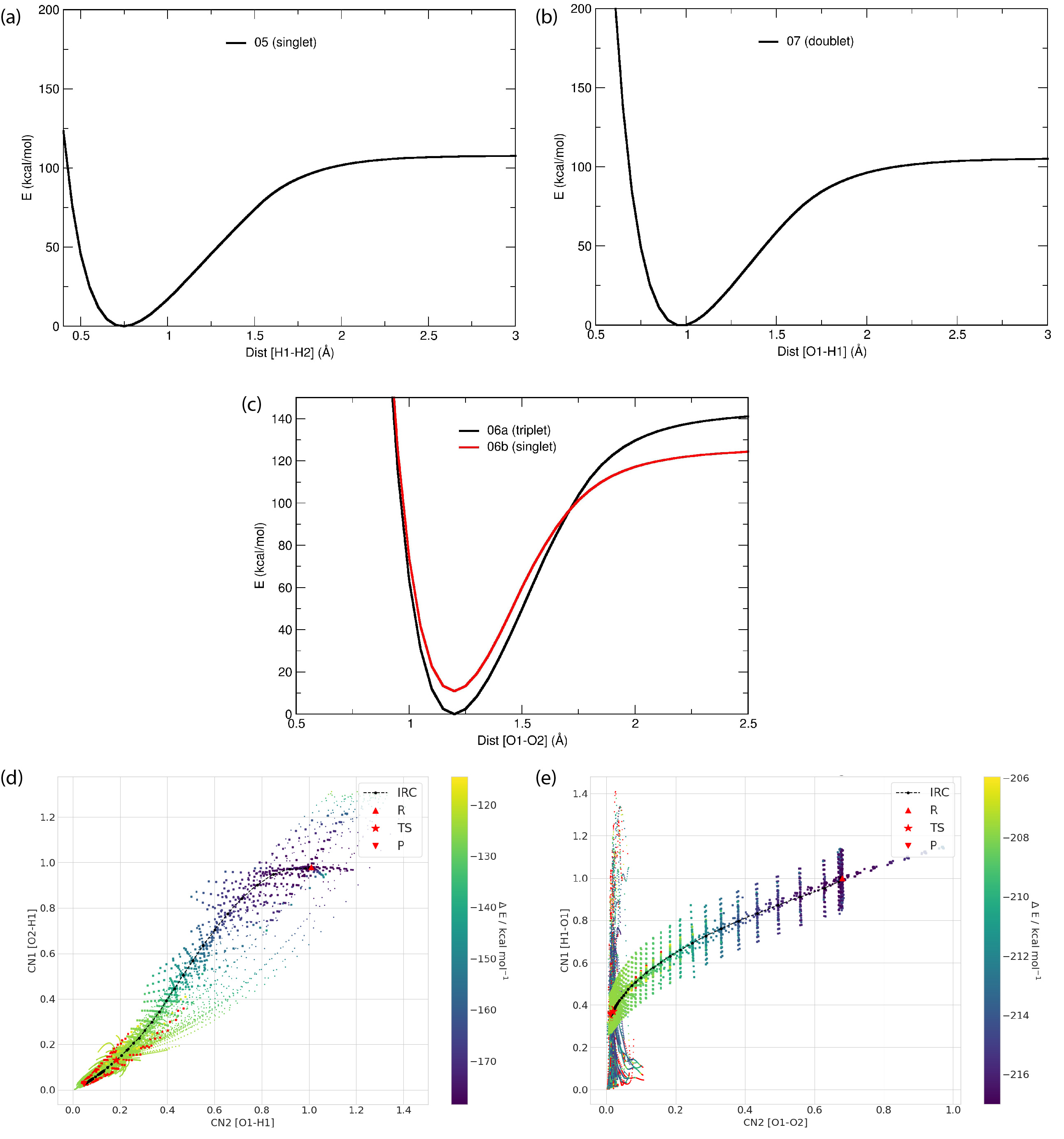

Supplement: Supplementary file 1 — Fig S1 [file 41597_2022_1330_MOESM1_ESM.jpg]

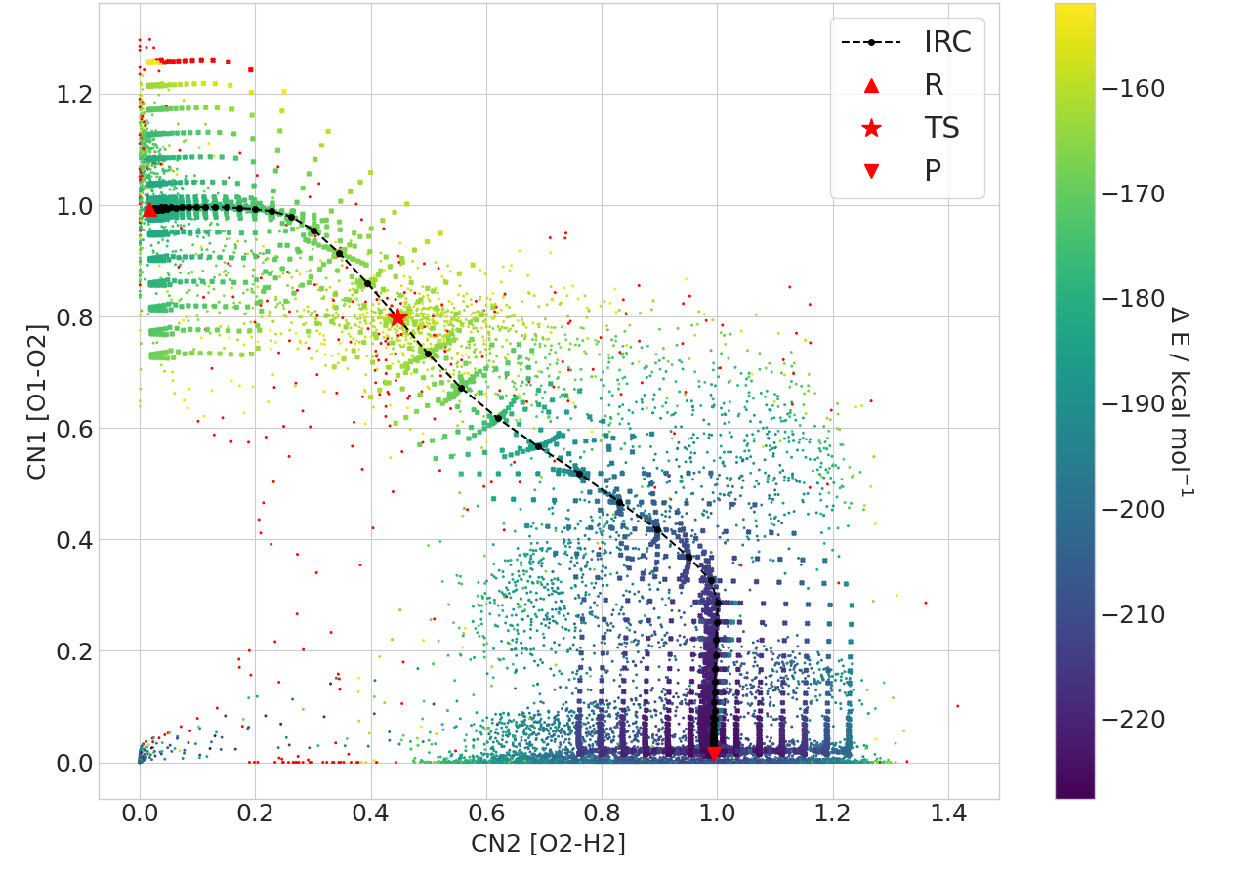

Supplement: Supplementary file 2 — Fig S2 [file 41597_2022_1330_MOESM2_ESM.jpg]

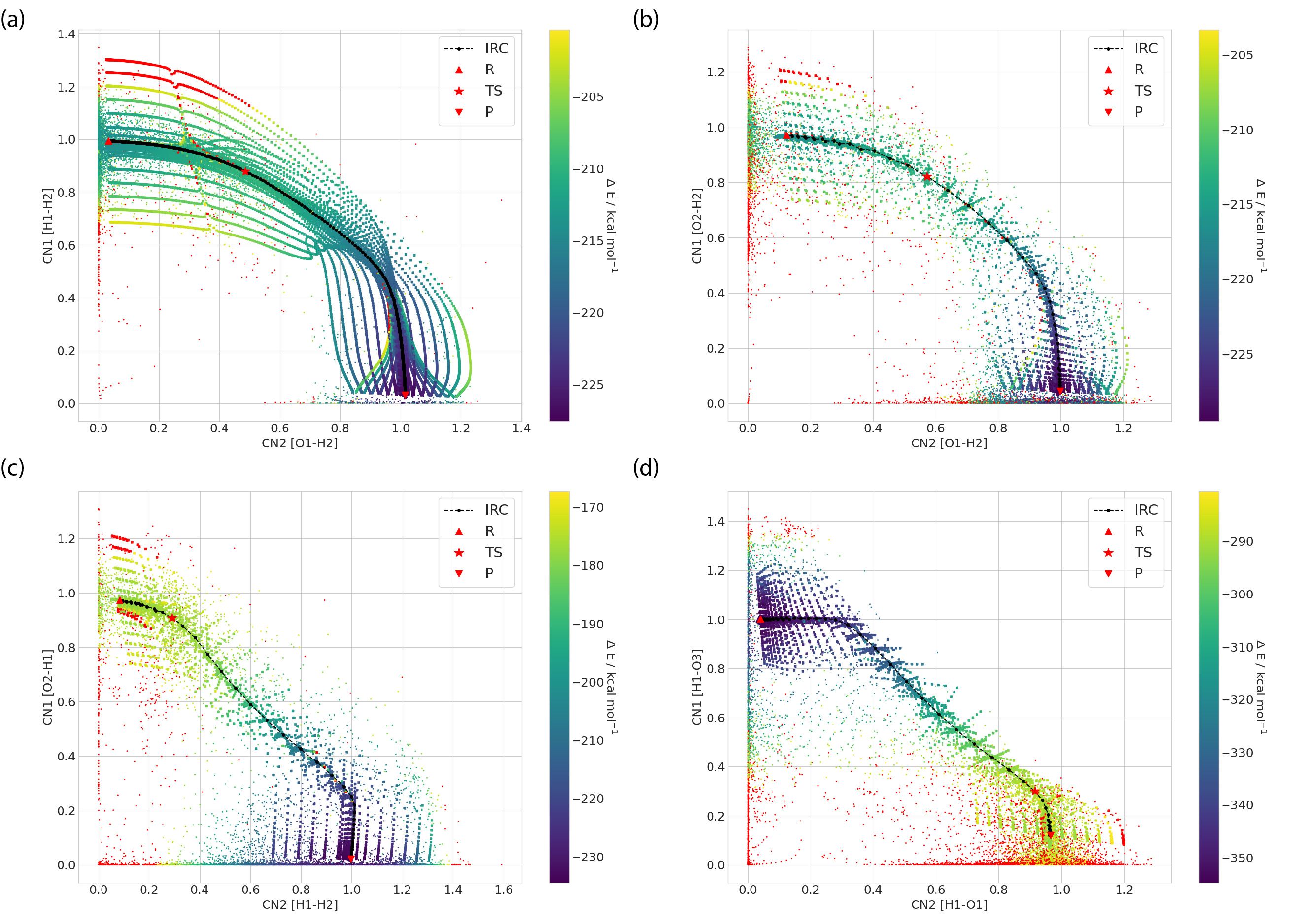

Supplement: Supplementary file 3 — Fig S3 [file 41597_2022_1330_MOESM3_ESM.jpg]

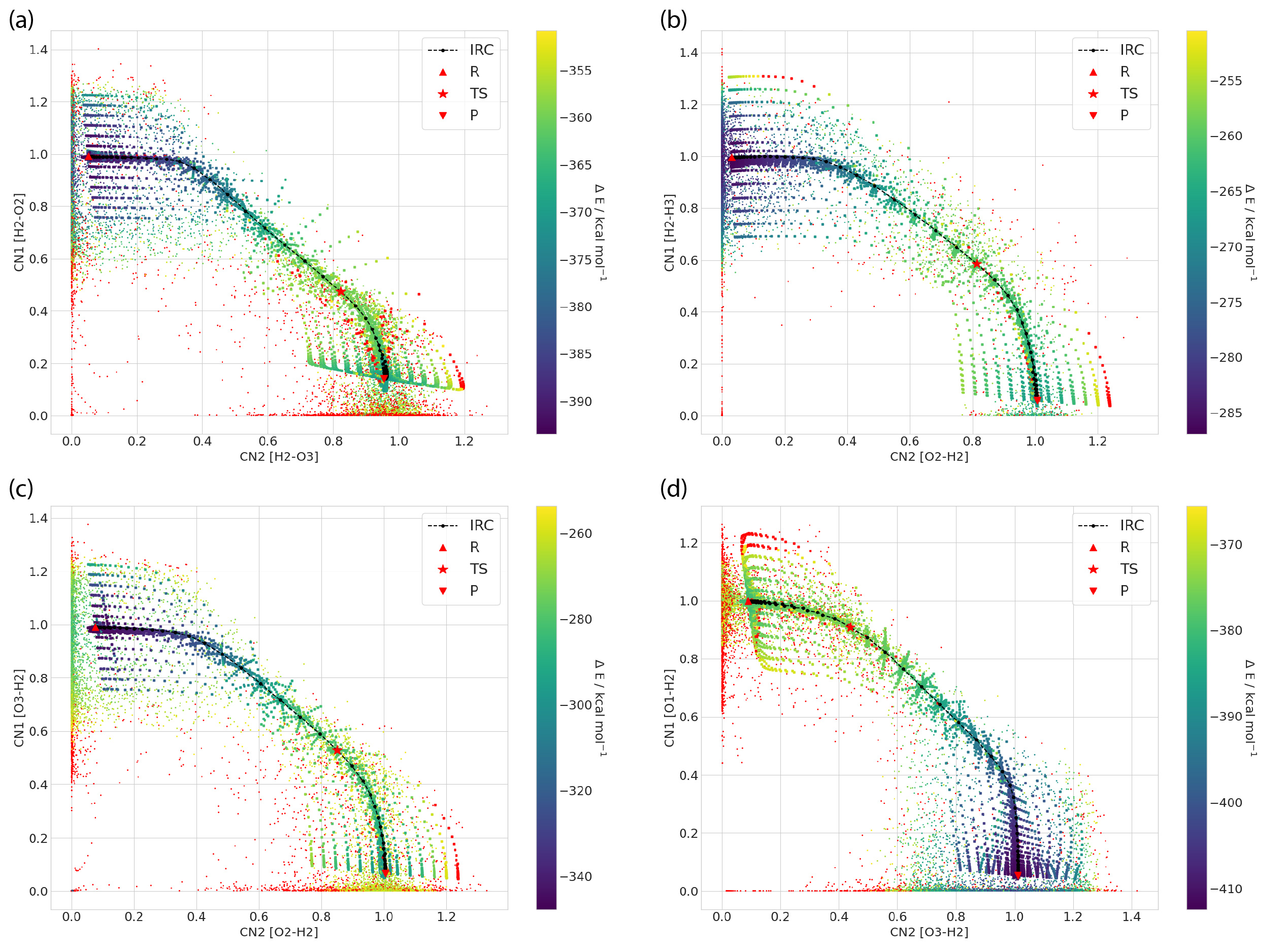

Supplement: Supplementary file 4 — Fig S4 [file 41597_2022_1330_MOESM4_ESM.jpg]
